# Smoothing a reference pathway based on discrete exogenous projections

**DOI:** 10.1016/j.mex.2021.101605

**Published:** 2021-12-14

**Authors:** Taoyuan Wei

**Affiliations:** CICERO Center for International Climate Research, P.O. Box 1129, Blindern, NO-0318 Oslo, Norway

**Keywords:** Computable general equilibrium, Scenario analysis, Reference scenario, Business-as-usual scenario, Economic growth, Energy supply and consumption, Integrated assessment, Calibration variables, CGE, Computable general equilibrium, BAU, Business-as-usual

## Abstract

In integrated assessment studies and scenario analysis, typically we need to calibrate a model to follow an exogenous pathway, e.g., the shared socioeconomic pathways. In these exogenous pathways, the data of key variables such as GDP and energy consumption are typically provided every five or ten years. In some cases, we need a yearly smooth pathway that is consistent with such an exogenous pathway with data of only every five years. Hence, this piece of study provides a method to obtain a smooth yearly pathway based on exogenous data of limited years.

• *An approach to smoothing time series of discrete variables is provided*
• *An example is used to illustrate the approach based on the data in World Energy Outlook 2019*

## Graphical abstract

**Figure:**
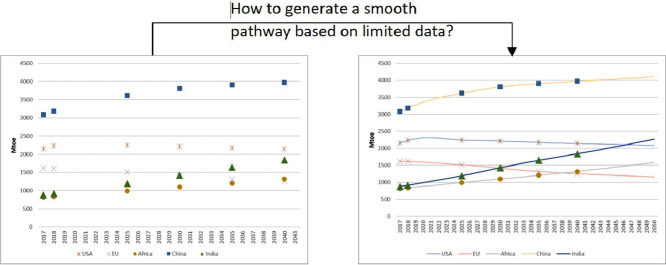

## Specifications table

**Table:** 

Subject Area:	Environmental Science
More specific subject area:	*Integrated assessment modeling; scenario analysis; energy demand and consumption; economic growth pathway; computable general equilibrium (CGE)*
Method name:	*Smoothing a reference pathway based on discrete exogenous projections*
Name and reference of original method:	*Not applicable.*
Resource availability:	*Not applicable.*

## Method details

In integrated assessment modeling for scenario analysis (e.g. [1, 3]), a reference or business-as-usual (BAU) scenario is typically necessary to serve as a basis to figure out the differences between alternative scenarios. A common way to build up a BAU scenario is calibrating a model to follow an exogenous pathway, e.g., the shared socioeconomic pathways [4] and the energy scenarios presented in annual world energy output reports by International Energy Agency (e.g. [2]). In these exogenous pathways, the data of key variables such as GDP and energy consumption are typically provided every five or ten years rather than yearly. In some cases, to simulate a scenario without a sudden jump from one year to another in a model, we prefer a yearly smooth pathway consistent with such an exogenous pathway with data of only every five years. To the best of my knowledge, although some modelers have done similar work, no study has formally presented an approach to do such a job and thus this piece of study provides a method to obtain a smooth yearly pathway based on exogenous data of limited years. The approach described below is based on the data of the Stated Policy Scenario (STEPS) provided in the Appendix A of World Energy Outlook 2019 [2]. Below we take total primary energy demand (TPED) data as an example to illustrate the approach.

**Fig. 1:**
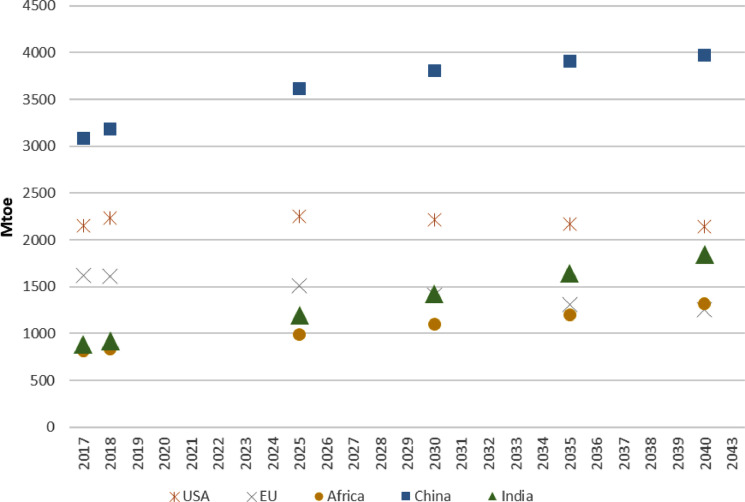
The total primary energy demand (TPED) of five regions in the Stated Policy Scenario (STEPS) presented by IEA [2]. Unit: Mtoe.

In IEA [2], total primary energy demand (TPED) data for one scenario are for various regions and countries, including North America, United States, Central and South America, Brazil, Europe, European Union, Africa, South Africa, Middle East, Eurasia, Russia, Asia Pacific, China, India, Japan, and Southeast Asia. The data of each region/country are available only for specific years of 2017, 2018, 2025, 2030, 2035, and 2040. Fig. 1 shows TPED for five of these regions. In this study, we use a mathematical approach to calibrating a future development path of TPED for each of the regions until 2050 based on the limited data from IEA [2] as illustrated by Fig. 1.

A smooth pathway of a region based on the known TPED data of limited years must pass each of the known TPED data. A smooth pathway also implies that the growth rates derived from the pathway before and after a joint point should be the same. Below an approach is developed to calibrate a smooth pathway of a region that satisfies both conditions.

For each time span of 2018–2025, 2025–2030, 2030–2035, 2035–2040, and 2040–2050, we assume that the TPED in a region follows an exponential path,

(1) $${\hat{E}}_{t}\left(t_{0}\right)={\hat{E}}_{t_{0}}\left(t_{0}\right)e^{\left[\frac{1}{3}a\left(t_{0}\right){\left(t-t_{0}\right)}^{3}+\frac{1}{2}b\left(t_{0}\right){\left(t-t_{0}\right)}^{2}+c\left(t_{0}\right)\left(t-t_{0}\right)\right]},\;t_{0}=2018,\;2025,\;2030,\;2035\;or\;2040$$

where ${\hat{E}}_{t}\left(t_{0}\right)$ is the calibrated TPED at the year t based on the TPED at the year $t_{0}$; ${\hat{E}}_{t_{0}}\left(t_{0}\right)=E_{t_{0}}$, which is the known TPED at the year $t_{0}$ provided by IEA [2]; $a(t_{0})$, $b(t_{0})$, and $c(t_{0})$ are parameters to be calibrated. This smooth path corresponds to growth rates of the calibrated TPED,

(2) $$\begin{array}{ccc} {\hat{D}}_{t}\left(t_{0}\right) & = & \frac{\frac{d{\hat{E}}_{t}\left(t_{0}\right)}{dt}}{{\hat{E}}_{t}\left(t_{0}\right)}=a\left(t_{0}\right){\left(t-t_{0}\right)}^{2}+b\left(t_{0}\right)\left(t-t_{0}\right)+c\left(t_{0}\right),\\ t_{0} & = & 2018,\;2025,\;2030,\;\;2035,\;or\;2040 \end{array}$$

Since we do not know TPED of any year after 2040, particularly 2050, then we simply assume that yearly growth rates of 2040–2050 are constant, i.e., $a\left(t_{0}=2040\right)=\;b\left(t_{0}=2040\right)=0$. It is also possible to adopt other assumptions. The purpose here is to determine how TPED changes after 2040.

To obtain $\hat{D}$s, which determine the TPED path from 2018 to 2050, we have 13 parameters to be calibrated, i.e., $a(t_{0})$, $b(t_{0})$, and $c(t_{0})$ for $t_{0}=2018,\;2025,\;2030,\;2035,\;or\;2040$ except $a\left(t_{0}=2040\right)=\;b\left(t_{0}=2040\right)=0$.

We assume the path of the growth rates is smooth so that the growth rate of any joint year of 2025, 2030, 2035, and 2040 can be obtained from the formula of both time spans before and after the specific years. For example, the TPED growth rate in the year 2025 satisfies

(3) $${\hat{D}}_{t=2025}\left(t_{0}=2018\right)={\hat{D}}_{t=2025}\left(t_{0}=2025\right)$$

Here we have 4 independent equations corresponding to the four joint years.

In addition, at each of the joint years 2025, 2030, 2035, and 2040, we assume a smooth change in the growth rate, which implies that the derivatives of growth rates at each of the joint years are equalized for both time spans before and after the joint year. Still taking the year 2025 as an example, we have

(4) $$2a\left(t_{0}\right)\left(t-t_{0}\right)+b\left(t_{0}\right)=b\left(t\right),\;t_{0}=2018,\;t=2025$$

Here we have 4 independent equations corresponding to the years 2025, 2030, 2035, and 2040.

Since we know the TPED data for both 2017 and 2018, we also force the growth in 2018 to satisfy

(5) $$1+{\hat{D}}_{t=2018}\left(t_{0}=2018\right)=E_{2018}/E_{2017}$$

Where $E_{2018}$ and $E_{2017}$ are the TPED data directly taken from IEA [2].

Similarly as we know the TPED data at the years 2018, 2025, 2030, 2035, and 2040 directly from IEA [2], we can calculate the changes in each of the periods: 2018–2025, 2025–2030, 2030–2035, and 2035–2040. Then we have, for example, for the period 2025–2030,

(6) $$\prod_{t=2026}^{2030}\left(1+{\hat{D}}_{t}\left(t_{0}=2025\right)\right)=E_{t=2030}/E_{t=2025}$$

Here we have another 5 independent questions corresponding to the known yearly growth rate in 2018 and the four time spans.

**Fig. 2:**
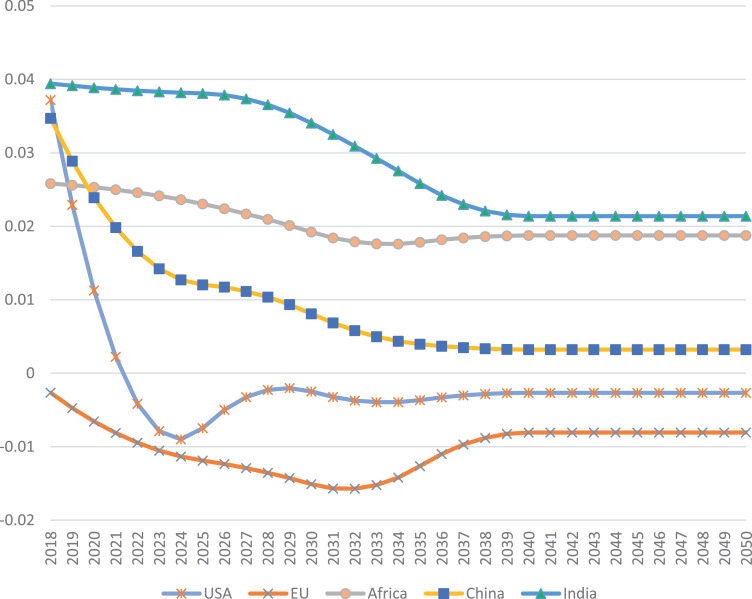
The calibrated yearly growth rates of the total primary energy demand (TPED) of five regions in the Stated Policy Scenario (STEPS) presented by IEA [2]. Unit: percent.

Hence, we solve the 13 questions simultaneously to calculate the unknown 13 parameters to determine the growth rates of each year from 2018 to 2050. The calibrated parameters for the five regions in Fig. 1 are shown in Table 1. The calibrated parameters are very different from one year to another, indicating dramatic changes in TPED from one time span to another. Based on the calibrated parameters, the growth rates of TPED of a region can be calculated out from Eq. (2). Fig. 2 shows the growth rates of TPED for the five regions in Fig. 1. Our results can capture the different growth rates across time spans. For example in EU, rather than a continuously decreasing curve, the growth rates drop dramatically at the beginning years and then increase slightly before become quite stable after 2030.

**Table 1:** Calibrated parameters for the smooth pathway of TPED.

Parameter	Year	USA	EU	Africa	China	India
a	2018	0.001319	0.000128	−3.10E-05	0.00043	1.57E-05
a	2025	−0.00037	−4.3E-05	−3.06E-05	−0.00011	−1.45E-04
a	2030	0.000125	0.000268	1.28E-04	0.000106	−2.34E-05
a	2035	−3.9E-05	−0.00018	−3.63E-05	2.98E-05	1.77E-04
b	2018	−0.01562	−0.00222	−1.77E-04	−0.00625	−2.99E-04
b	2025	0.002848	−0.00042	−6.12E-04	−0.00022	−7.90E-05
b	2030	−0.00086	−0.00085	−9.18E-04	−0.00136	−1.53E-03
b	2035	0.000394	0.001831	3.63E-04	−0.0003	−1.77E-03
c	2018	0.037212	−0.00265	2.58E-02	0.034695	3.94E-02
c	2025	−0.00747	−0.01189	2.30E-02	0.012055	3.81E-02
c	2030	−0.0025	−0.01508	1.92E-02	0.008105	3.41E-02
c	2035	−0.00365	−0.01264	1.78E-02	0.003966	2.58E-02
c	2040	−0.00266	−0.00806	1.87E-02	0.003222	2.14E-02

**Fig. 3:**
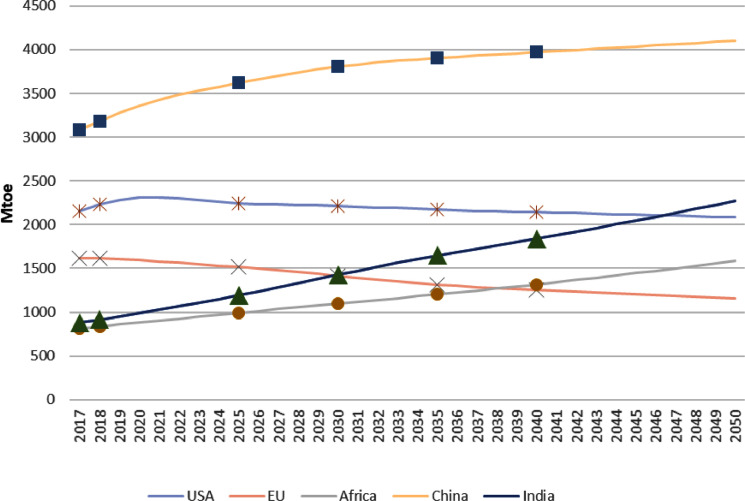
The calibrated smooth path of the total primary energy demand (TPED) of five regions, which pass the corresponding data in the Stated Policy Scenario (STEPS) presented by IEA [2]. Unit: Mtoe.

The estimated yearly growth rates are used to calculate the yearly TPED for a region/country in IEA [2]. For the five regions shown in Fig. 1, the calibrated yearly TPEDs are shown in Fig. 3. Compared to the growth rates in Fig. 2, the changes in TPEDs over time tend to be much more stable.

**Fig. 4:**
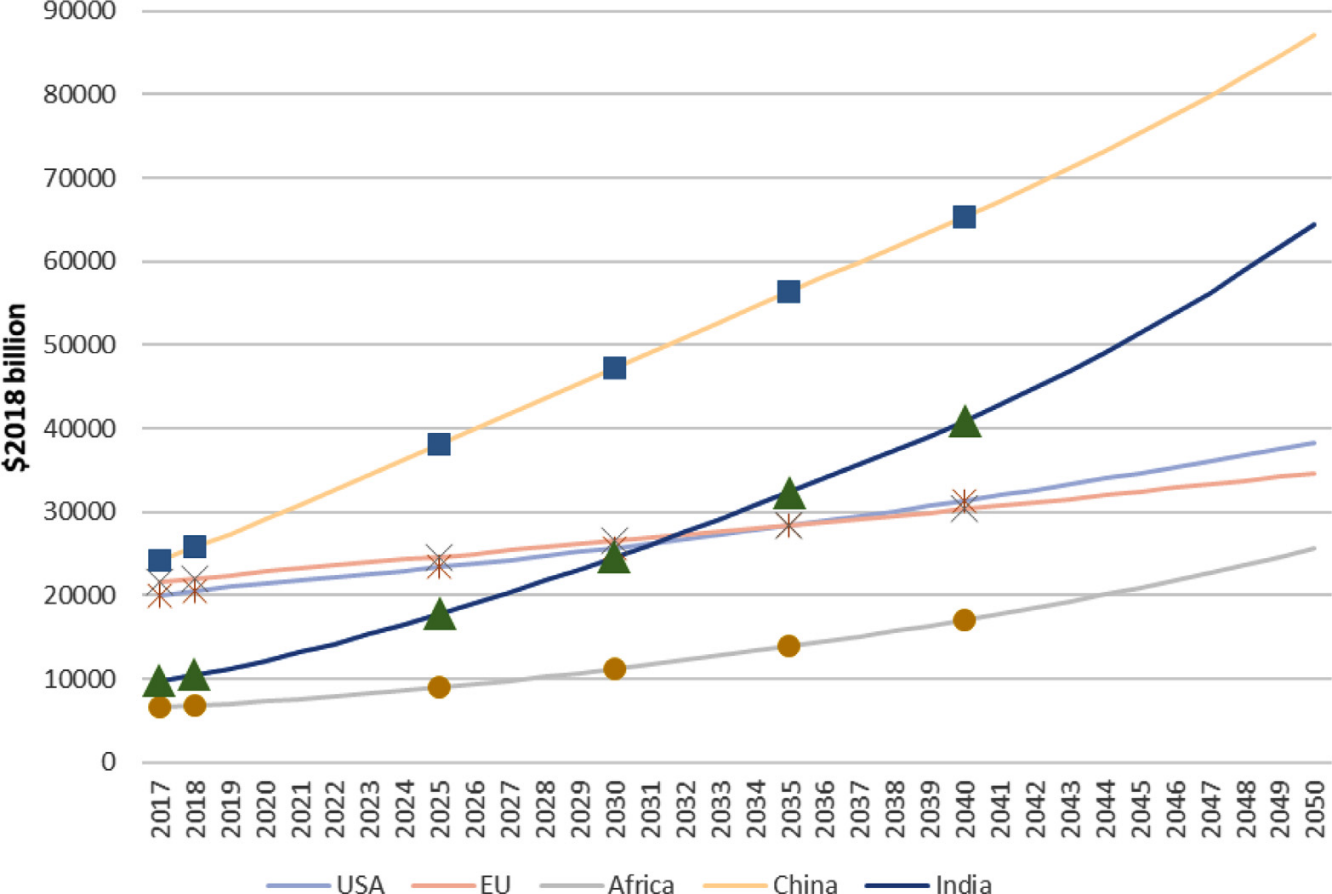
The calibrated smooth path of the gross domestic product (GDP) of five regions, which pass the corresponding data in the Stated Policy Scenario (STEPS) presented by IEA [2]. Unit: $2018 billion.

**Fig. 5:**
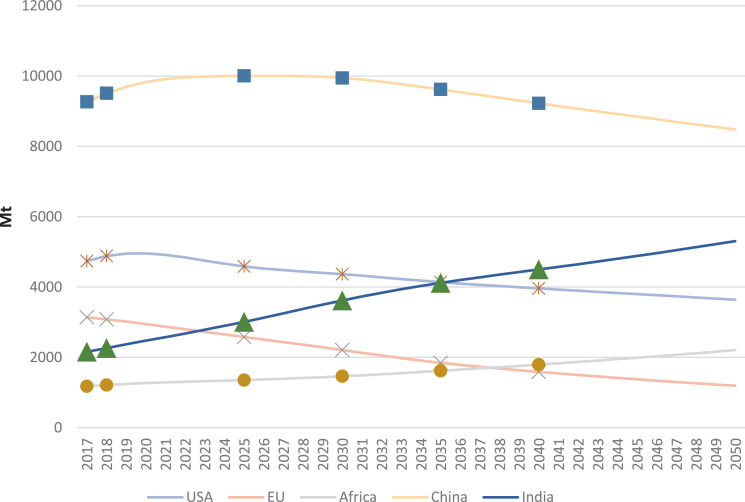
The calibrated smooth path of the carbon dioxide (CO_2_) emissions of five regions, which pass the corresponding data in the Stated Policy Scenario (STEPS) presented by IEA [2]. Unit: Mt.

Similarly, the other variables reported in IEA [2] such as gross domestic product (GDP) and CO2 emissions can also be treated in the same way. Fig. 4, Fig. 5 show respectively the generated smooth paths of GDP and CO2 emissions for the selected five regions. All the data and codes written in GAMS (www.gams.com) for this study are provided as online supplementary information.

## Declaration of interests

The author declares that he has no known competing financial interests or personal relationships that could have appeared to influence the work reported in this paper.

## Appendix Supplementary materials

Image, application 1

Image, application 2

Image, application 3

Image, application 4
